# COVID-19 Vaccine Hesitancy and Psychosocial Effects of the COVID-19 Pandemic among Health-Science Students of Lithuania–A National Cross-Sectional Online Survey

**DOI:** 10.3390/ijerph182412870

**Published:** 2021-12-07

**Authors:** Jonas Montvidas, Milda Basevičiūtė, Kamilė Burokaitė, Virginija Adomaitienė, Sigita Lesinskienė

**Affiliations:** 1 Psychiatry Department, Medical Faculty, Medical Academy, Lithuanian University of Health Sciences, LT-44307 Kaunas, Lithuania; milda.baseviciute@stud.lsmu.lt (M.B.); virginija.adomaitiene@kaunoklinikos.lt (V.A.); 2 Psychiatry Department, Faculty of Medicine, Institute of Clinical Medicine, Vilnius University, LT-01513 Vilnius, Lithuania; kamile.burokaite@mf.stud.vu.lt (K.B.); sigita.lesinskiene@mf.vu.lt (S.L.)

**Keywords:** COVID-19, vaccine hesitancy, health-science students

## Abstract

(1) Background: the relationship between the psychosocial effects of the COVID-19 pandemic and COVID-19 vaccine hesitancy is understudied. Moreover, health science students are the future leaders and advocates of vaccination efforts. Therefore, it is essential to understand the origins of vaccine hesitancy and evaluate if the adverse psychosocial effects of the COVID-19 pandemic influence it. (2) Methods: we shared an anonymous questionnaire among health-science students via institutional emails of two Lithuanian universities. Results were summarized with odds ratios and mean differences. (3) Results: a total of 1545 health sciences students answered the questionnaire. Almost a fifth of the respondents claimed that they were unsure about getting vaccinated, and nearly one out of ten claimed that they would not get vaccinated. Medicine students, non-infected students, and students who volunteered in a COVID-19 ward were significantly more willing to get vaccinated compared to other health science students. Vaccine hesitant respondents reported a more significant negative effect of COVID-19 on their income and belief in the future. (4) Conclusions: the results of this study showed that negative psychosocial impact on income and ‘belief in future’ were positively associated with vaccine hesitancy. Having been diagnosed with COVID-19 was significantly associated with being doubtful towards vaccination.

## 1. Introduction

The World Health Organization (WHO) declared the severe acute respiratory syndrome coronavirus 2 (SARS-COV2, COVID-19) infection a global pandemic on the 11 March 2020 [1]. According to WHO, there were over 130 million confirmed cases and 2.8 million confirmed deaths in 223 countries on the 5 April 2021 [2]. Without specific treatment options, social distancing and national quarantines were imposed to control the spread of this pathogen. However, these measures had significant flaws such as economic burden and a negative impact on mental health, making them unsuitable for prolonged use [3,4,5]. Recognizing the need for other options to control the pandemic, unprecedented efforts were made to create COVID-19 vaccines.

However, vaccine hesitancy remains a significant obstacle in immunization efforts [6]. MacDonald et al. described this phenomenon as a “delay in acceptance or refusal of vaccination despite the availability of vaccination services” [7]. Several 2020 studies found that only 49–70% of respondents from the United States of America (USA) considered getting vaccinated against COVID-19 as soon as a vaccine was available [8,9]. Lazarus et al. found that vaccine acceptance varied considerably between countries, with the lowest seen in Russia, where only 55% of respondents claimed that they would get vaccinated. The highest was found in China, where 90% of respondents said they would get vaccinated [10].

Various studies were performed to identify the main reasons for vaccine hesitancy. Age was positively related to vaccine acceptance, with the older population being more willing to get vaccinated [9]. Lack of knowledge about vaccines was another risk factor analyzed, and educational efforts showed a beneficial effect on the attitudes towards vaccination. Doubts regarding the safety of the vaccines was another reason for hesitating [10]. On the other hand, we found only a few studies looking into the relationship between the psychosocial effects of the COVID-19 pandemic and attitude towards vaccination. Moreover, we did not find studies comparing the psychological effects of quarantine on the acceptance of vaccines against COVID-19.

One might think that rates of vaccine hesitancy among health science students should be very low. However, vaccine hesitancy is prevalent among health science students. An Italian study performed by Barello et al. found that one out of ten medical students was reluctant to get vaccinated against COVID-19. No significant difference in attitudes towards vaccination was found between medical and non-medical students [11]. In a Maltese sample of Health Sciences students, 30% answered that they were unlikely to take the COVID-19 vaccine [12]. Saied et al. found that 46% of an Egyptian medical student’s sample were hesitant towards the COVID-19 vaccine [13]. In a study performed in the United States of America, 23% of medical students answered that they would not take the COVID-19 vaccine immediately upon FDA approval [14]. However, none of these studies evaluated the relationship between the psychosocial effects of the COVID-19 pandemic and vaccine hesitancy.

Health science students will be responsible for future vaccination efforts. Therefore, it is vital to understand the reasons behind vaccine hesitancy in this population. Moreover, the effects of the pandemic on the attitude towards vaccination have been heavily understudied. Recognizing the lack of studies evaluating the influence of the psychosocial impact of the COVID-19 pandemic on the attitude towards vaccination, we decided to perform a national cross-sectional survey of health science students in Lithuania. The primary outcome of this study was to determine whether adverse psychosocial effects of the COVID-19 pandemic were associated with the attitude towards vaccination against COVID-19.

## 2. Materials and Methods

We shared Lithuanian and English versions of an anonymous survey via institutional emails of Lithuania’s two largest health science universities—the Lithuanian University of Health Sciences (LUHS) and Vilnius University (VU). Participants were informed about the aims and scope of the survey and answered the questions of their own free will.

Inclusion criteria were being an undergraduate health science (medicine, dentistry, nursing, public health, physiotherapy, occupational therapy, or pharmacy) student in either LUHS or VU during the time of data collection (from 1 February 2021 until 31 March 2021).

The online survey consisted of the COVID-19 questionnaire for students developed by prof. Andre Sourander from Turku University, Finland (Table 1). The first four questions asked about socio-demographic data. Questions five to seven asked about COVID-19 infection status, vaccination status, and whether a person volunteered in a COVID-19 ward. Questions eight to fourteen aimed to evaluate whether the pandemic negatively affected the respondents financially, socially and academically. Questions fifteen to nineteen aimed to assess how severe was the stress, anxiety, depressed mood and other negative feelings caused by the adverse psychosocial effects of the pandemic. Respondents marked the answers to questions from eight to nineteen on a 5-point Likert scale with one meaning “had no effect” and five “had a strong negative effect”. We calculated the global score of questions eight to nineteen by adding the points (1–5) together.

We used the Statistical Package for the Social Sciences (SPSS) version 27 (IBM, Chicago, IL, USA) for the statistical analysis. Cronbach’s alpha was calculated to determine the internal consistency of questions number eight to 19. Multinomial logistic regression was used to calculate the odds ratios. We used study disciple as a factor variable, and infection status as a dependent variable to calculate the odds ratios for medical students being infected with COVID-19 compared to other health science students. Multinomial logistic regression was also performed to calculate the odds ratios for getting vaccinated. Study disciple, infection status, and volunteering were used as factor variables, and vaccination status was used as a dependent variable. Mean and total scores of the Likert scales were calculated. One-way ANOVA with Tukey post hoc multivariate comparisons was calculated to check if the mean scores of the Likert scales differed between the respondents based on their intent to get vaccinated. The Likert scale scores were used as dependent variables, and vaccination status was used as a factor variable. The level of significance was kept at 0.05.

## 3. Results

### 3.1. Demographic Data

A total of 1545 health sciences students answered the questionnaire (Table 2). Medicine students made up 62.5% (*n* = 965) of the sample. We summated other health science students into one group, which made up the remaining 37.5% (*n* = 580) of the sample. Most respondents were Lithuanian (86.2%, *n* = 1332). More than two-thirds of the respondents (71.4%, *n* = 1103) had not been infected, while 11.8% (*n* = 183) reported that they had been infected with SARS-COV2. Those who volunteered made up 13.1% (*n* = 202) of the sample. Most respondents answered that they are planning to get vaccinated against SARS-COV2 (72.6%, *n* = 1121), while almost a fifth (19.2%, *n* = 296) claimed that they were unsure about getting vaccinated, and almost one out of 10 (8.3%, *n* = 128) claimed that they will not get vaccinated.

### 3.2. Association between Study Discipline, Volunteering, Infection Status and Vaccine Acceptance

Medicine students were significantly less likely to be infected compared to students of other health sciences (OR = 0.64, 95% CI 0.47–0.88, *p* = 0.006). Moreover, medicine students were significantly more likely to have volunteered (OR = 2.39, 95% OR 1.68–3.39, *p* < 0.001) or to be willing to get vaccinated (OR = 1.7, 95% CI 1.18–2.46, *p* = 0.005) compared to students of other health sciences. Students who were not infected with SARS-COV2 were significantly more pro vaccination compared to those students who were diagnosed with COVID-19 (OR = 1.9, 95% CI 1.37–2.65, *p* < 0.001) or those students who were unsure about their infection status (OR = 2.07, 95% CI 1.56–2.76, *p* < 0.001). Students who volunteered where significantly more likely to get vaccinated (OR = 2.5, 95% CI 1.51–3.97, *p* < 0.001).

### 3.3. The Mean Scores of the Questions Evaluating the Negative Psychosocial Impact of the COVID-19 Pandemic and the Negative Feelings Associated with the Negative Psychosocial Impact of the COVID-19 Pandemic

The respondents reported the most significant negative psychosocial impact on studies (m = 3.7, SD 1.3) and friendships (m = 3.11, SD 1.29). The mean scores for the negative psychosocial impact of the COVID-19 pandemic are listed in Table 3.

The respondents reported having experienced the most severe negative feelings associated with the negative psychosocial effect of COVID-19 on studies (m = 3.57, SD 1.39) and the belief in future (m = 3.04, SD 1.5). The mean scores of the negative feelings associated with the negative psychosocial impact of the COVID-19 pandemic are listed in Table 4.

### 3.4. Differences of Mean Scores of the Negative Psychosocial Impact of the COVID-19 Pandemic and the Negative Feelings Associated with the Negative Psychosocial Impact of the COVID-19 Pandemic between Respondents Based on their Intent to Get Vaccinated

Respondents who reported that they were not planning to get vaccinated (difference of means = 0.37, 95% CI 0.05–0.69, *p* = 0.02) or were unsure about vaccination (difference of means = 0.27, 95% CI 0.05–0.49) reported significantly greater negative impact of the COVID-19 pandemic on income and economy. Students who were not planning to get vaccinated reported significantly more negative feelings associated with the negative impact of the COVID-19 pandemic on their income and economy than those who were planning to get vaccinated (difference of means = 0.41, 95% 0.09–0.73, *p* = 0.003). Moreover, students who were not planning to get vaccinated reported significantly greater negative impact on their belief in future compared to those who were planning to get vaccinated (difference of means = 0.37, 95% CI 0.05–0.68, *p* = 0.02) or were unsure (difference of means = 0.4, 95% CI 0.05–0.76, *p* = 0.02). However, respondents who stated that they are planning to get vaccinated reported a greater negative impact of the COVID-19 pandemic on their belief in the future than those who were unsure about vaccination (difference in means = 0.21, 9% CI 0.05–0.68, *p* = 0.01). The significant differences in mean scores of the Likert scales are listed in Table 5. The means of the total scores of the negative psychosocial impact of the COVID-19 pandemic differed statistically significantly between the respondents who were going to get vaccinated (m = 17.57, SD 5.23) and those who were not going to get vaccinated (m = 18.68, SD 5.84). Total Likert scale mean scores did not differ statistically significantly in any other group.

## 4. Discussion

In our sample of health science students from seven different health science fields studying in Lithuania’s two largest health science universities, we found that health science students who were not planning to get vaccinated reported the most significant negative impact of the COVID-19 pandemic on income and economy. In contrast, students who were unsure about vaccination reported a more significant impact on income and economy than students planning to get vaccinated. Similarly, Bertoncello et al. found that lower income was associated with greater vaccine hesitancy in an Italian sample [15]. Guay et al. also reported that low to moderate income was related to greater vaccine hesitancy in a sample from Quebec, United States of America [16]. These examples support the association between income and vaccine hesitancy. However, it is essential to note that, findings of our study highlight that health science students suffering more significant effects on their income rather than having lower income in general are more prone to be vaccine-hesitant.

Respondents that were not planning to get vaccinated reported a greater negative effect of the COVID-19 pandemic on their ‘belief in future’ compared to both the students claiming that they would get vaccinated and those who were hesitant. We could not find other studies evaluating the association between general optimism about the future and vaccine acceptance. However, we found numerous articles examining the relationship between conspiracy beliefs and willingness to get vaccinated. For example, Pivetti et al. found that believing in conspiracy theories was a negative predictor of COVID-19 vaccine hesitancy [17]. These results were replicated in various other studies, showing an inverse correlation between believing in conspiracies and intention to get vaccinated either in a present time or in the future [18]. Similar results were also produced by Bierwiaczonek et al. They found that believing in COVID-19 related conspiracy theories was associated with poorer adherence to social distancing rules [19]. Salali et al. found that believing that the coronavirus infection was not created in a lab, but was a natural disease, increased the likelihood of vaccination [20]. Goldberg et al. even found that anti-vaccination beliefs were highly correlated to anecdotal misconceptions that the former president of the United States of America was a Muslim and other conspiracies [21]. These examples illustrate the association between the general state of unstable beliefs and expectations of the future and vaccine hesitancy. We believe that this association merits further investigation.

We found that medical students were significantly more pro-vaccination compared to other health science students. In our opinion, this could be explained by more excellent knowledge about vaccination among medical students compared to other health science students [22]. Rostkowska et al. demonstrated that medical students have good knowledge about vaccines, and it was shown in various studies that poorer education is a risk factor for vaccine hesitancy [15,23]. For example, Malik et al. found that level of education and age and gender were among the most specific predictors of vaccine hesitancy [24]. Also, specific knowledge about vaccines might increase the percentage of those willing to get vaccinated, as was shown by Logan et al. [25]. Therefore, our findings further proved that knowledge about vaccines is a significant risk factor of vaccine hesitancy.

Another reason why medical students were more likely to get vaccinated could be since they had closer contact with COVID-19 patients as they were also significantly more likely to volunteer in a COVID-19 ward. We found no studies investigating the association between volunteering in COVID-19 wards and vaccine acceptance. However, healthcare professionals caring for COVID patients report greater vaccine acceptance [26,27]. On the other hand, healthcare workers who were not working in COVID wards showed vaccine acceptance somewhat like the general public [28,29]. Therefore, we believe that our findings illustrate that direct contact with COVID-19 is a distinct positive predictor of vaccine acceptance.

Health science students who claimed to have been infected with COVID-19 showed significantly higher vaccine disapproval than other groups. We could not find many studies evaluating the association between having recovered from COVID-19 and vaccine acceptance. However, a study performed in Italy by Gerussi et al. found that being hospitalized during the acute phase of COVID-19 infection did not increase the acceptance of the COVID-19 vaccine, with only 28% of respondents claiming that they are willing to take the COVID-19 vaccine [30]. We believe that the relationship between being infected with COVID, and vaccine hesitancy needs further investigation.

Finally, the main benefit of this study is that this is the first study investigating the association between the scope of the psychosocial impact of the COVID-19 pandemic and attitudes toward vaccination among health science students. The main drawback of this study was that there was no way to control who answered the survey. Also, we could have included more specific questions regarding vaccine acceptance, such as the reason for being against vaccination, willingness to get vaccinated once there was more data about safety and efficacy, belief in conspiracy theories which would have permitted us to get a more precise conclusion regarding vaccine hesitancy in our sample.

## 5. Conclusions

The results of this study showed that the negative psychosocial impact of the COVID-19 pandemic on income and ‘belief in future’ was positively associated with greater COVID-19 vaccine hesitancy. However, we did not find a significant association with the negative psychosocial impact of the COVID-19 pandemic on family relationships, friendships, studies, or health associated with attitudes towards vaccination. We also found that medical students were more willing to get vaccinated compared to other health science students. However, COVID-19 vaccine hesitancy was still prominent, with almost a fifth of the sample being unsure if they would get vaccinated against COVID-19. Being diagnosed with COVID-19 was significantly associated with being negligent towards COVID-19 vaccination and having volunteered was associated with vaccine acceptance.

We could not find other studies evaluating the association between optimism about the future or having been diagnosed with COVID-19 in the past and vaccine acceptance. Therefore, we believe these topics require further research.

## Figures and Tables

**Table 1:** Questionnaire of the national survey of the negative effect of the COVID-19 pandemic on the psychosocial well-being of health science students of Lithuania.

Question Number	Question	Answer Options
1	What is your gender?	Male Female Other
2	What is your university affiliation?	Vilnius University Lithuanian University of Health Sciences
3	What discipline do you study?	Medicine Dentistry Pharmacy Public health Nursing Physiotherapy Occupational therapy
4	What is your year of study?	1–6
5	Have you been infected with SARS-COV?	Yes No Unsure
6	Will you get vaccinated/have you already received the vaccine against COVID-19?	Yes No Unsure
7	Have you volunteered in a COVID-19 ward?	Yes No
8	How strongly did the COVID-19 pandemic negatively affect your friendships?	1 (had no effect) to 5 (had a strong negative effect)
9	How strongly did the COVID-19 pandemic negatively affect your family relationships?	1 (had no effect) to 5 (had a strong negative effect)
10	How strongly did the COVID-19 pandemic negatively affect your health?	1 (had no effect) to 5 (had a strong negative effect)
11	How strongly did the COVID-19 pandemic negatively affect your income and economy?	1 (had no effect) to 5 (had a strong negative effect)
12	How strongly did the COVID-19 pandemic negatively affect your studies?	1 (had no effect) to 5 (had a strong negative effect)
13	How strongly did the COVID-19 pandemic negatively affect your belief in the future?	1 (had no effect) to 5 (had a strong negative effect)
14	How severe were the negative feelings (stress, anxiety, depression) caused by the negative effect of COVID-19 on your friendships?	1 (no negative feelings) to 5 (severe negative feelings)
15	How severe were the negative feelings (stress, anxiety, depression) caused by the negative effect of COVID-19 on your family relationships?	1 (no negative feelings) to 5 (severe negative feelings)
16	How severe were the negative feelings (stress, anxiety, depression) caused by the negative effect of COVID-19 on your health?	1 (no negative feelings) to 5 (severe negative feelings)
17	How severe were the negative feelings (stress, anxiety, depression) caused by the negative effect of COVID-19 on your income and economy?	1 (no negative feelings) to 5 (severe negative feelings)
18	How severe were the negative feelings (stress, anxiety, depression) caused by the negative effect of COVID-19 on your studies?	1 (no negative feelings) to 5 (severe negative feelings)
19	How severe were the negative feelings (stress, anxiety, depression) caused by the negative effect of COVID-19 on your belief in the future?	1 (no negative feelings) to 5 (severe negative feelings)

**Table 2:** Demographic data.

Demographic Factor	Demographic Data
Gender	Male 15.1% (*n* = 234)
	Female 84.9% (*n* = 1311)
Nationality	Lithuanian 86.2% (*n* = 1332)
	International 13.8% (*n* = 213)
Study subject	Medicine 62.5% (*n* = 1332)
	Occupational Therapy 0.9% (*n* = 14)
	Physiotherapy 4.7% (*n* = 73)
	Nursing 7.8% (*n* = 121)
	Public Health 5.1% (*n* = 79)
	Pharmacy 8% (*n* = 123)
	Dentistry 11% (*n* = 170)
Have you been infected with SARS-COV2?	Yes 11.8% (*n* = 183)
	Unsure 16.8% (*n* = 259)
	No 71.4% (*n* = 1103)
Are you going to get vaccinated against SARS-COV2?	Yes 72.6% (*n* = 1121)
	Unsure 19.2% (*n* = 296)
	No 8.3% (*n* = 128)
Have you volunteered?	Yes 13.1% (*n* = 202)
	No 86.9% (*n* = 86.9%)

**Table 3:** Mean scores of the questions evaluating the negative psychosocial impact of the COVID-19 pandemic. SD, standard deviation.

Negative Psychosocial Impact on:	Mean Likert Scale Score	SD
Friendships	3.11	1.29
Family relationships	2.41	1.32
Health	2.85	1.32
Economy and income	2.55	1.46
Studies	3.7	1.3
The belief in future	3.05	1.44
Total score	17.67	5.32

**Table 4:** Mean scores of the questions evaluating the negative feelings associated to the negative psychosocial impact of the COVID-19 pandemic. SD, standard deviation.

Negative Feelings Associated with the Negative Psychosocial Impact on:	Mean Likert Scale Score	SD
Friendships	2.91	1.43
Family relationships	2.35	1.38
Health	2.74	1.41
Economy and income	2.49	1.47
Studies	3.57	1.39
The belief in future	3.04	1.5
Total score	17.1	6.22

**Table 5:** Association between the negative psychosocial impact of COVID-19 and vaccine acceptance. M, mean Likert scale score; SD, standard deviation.

Factor	Are You Going to Get Vaccinated? (A)	Are You Going to Get Vaccinated? (B)	Mean Difference (A-B)	*p* Value
Impact on Income and economy	Yes (m = 2.47, SD 1.44)	No (m = 2.84, SD 1.56)	−0.37 *	0.02
		Unsure (m = 2.47, SD 1.47)	−0.27 *	0.01
Impact on Studies	Yes (m = 3.73, SD 1.29)	No (me = 3.75, SD 1.36)	−0.02	0.99
		Unsure (m = 3.53, SD 1.3)	0.2 *	0.04
Impact on belief in future	Yes (m = 3.02, SD 1.43)	No (m = 3.39, SD 1.48)	−0.37 *	0.02
		Unsure (m = 2.99, SD 1.41)	0.03	0.91
Negative feelings associated to the impact on income and economy	Yes (m = 2.42, SD 1.45)	No (m = 2.83, SD 1.49)	−0.41 *	0.01
		Unsure (m = 2.63, SD 1.49)	−0.21	0.83

*p* value, significance level > 95%, * *p* < 0.05.

## Data Availability

Data is stored in the personal storage of the corresponding author and is not available online.
